# Capillarity ion concentration polarization as spontaneous desalting mechanism

**DOI:** 10.1038/ncomms11223

**Published:** 2016-04-01

**Authors:** Sungmin Park, Yeonsu Jung, Seok Young Son, Inhee Cho, Youngrok Cho, Hyomin Lee, Ho-Young Kim, Sung Jae Kim

**Affiliations:** 1 Department of Electrical and Computer Engineering, Seoul National University, Seoul 08826, South Korea; 2 Department of Mechanical and Aerospace Engineering, Seoul National University, Seoul 08826, South Korea; 3 Institute of Advanced Machines and Design, Seoul National University, Seoul 08826, South Korea; 4 Big Data Institute, Seoul National University, Seoul 08826, South Korea; 5 Inter-university Semiconductor Research Center, Seoul National University, Seoul 08826, South Korea

## Abstract

To overcome a world-wide water shortage problem, numerous desalination methods have been developed with state-of-the-art power efficiency. Here we propose a spontaneous desalting mechanism referred to as the capillarity ion concentration polarization. An ion-depletion zone is spontaneously formed near a nanoporous material by the permselective ion transportation driven by the capillarity of the material, in contrast to electrokinetic ion concentration polarization which achieves the same ion-depletion zone by an external d.c. bias. This capillarity ion concentration polarization device is shown to be capable of desalting an ambient electrolyte more than 90% without any external electrical power sources. Theoretical analysis for both static and transient conditions are conducted to characterize this phenomenon. These results indicate that the capillarity ion concentration polarization system can offer unique and economical approaches for a power-free water purification system.

## Abstract (web summary)

The great demand for freshwater for sustaining life on Earth calls for smart solutions to purify ocean water with minimal energy consumption. Here, Park *et al*. show a passive desalination method via ion concentration polarization generated by capillary-driven flow through ion-selective hydrogels.

## Graphical abstract

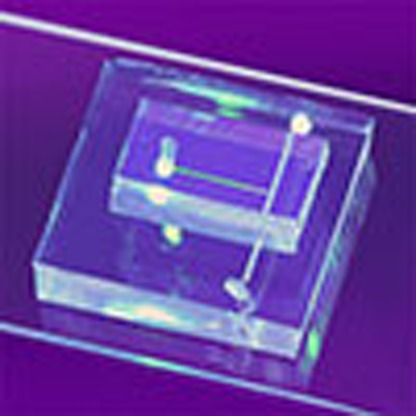

Recent noteworthy investigations in micro/nanofluidic devices enable to develop not only various biomedical but also significant energy and environmental applications^1^. Among these groundbreaking technologies, micro/nanofluidic platforms have successfully served as the differential shells of macro-scale water purification/desalination systems^2^,^3^,^4^. One can microscopically investigate the salt separation mechanism occurring near nanoporous membranes or porous electrodes by flow field tracking or voltage–current measurements. Although state-of-the-art desalination methods, such as reverse-osmosis and flash distillation, can provide the gigantic amount of fresh water with high energy efficiency^5^, low energy efficiency of small-scale platforms and the requirement of high-cost infrastructures have become acute nuisances, especially for remote/rural areas of the world. On the other hand, the micro/nanofluidic differential shells can be scalably integrated to a portable-scale system for individual usage or even to a mid-scale system for small village supply.

For such small-sized desalination/purification applications, ion concentration polarization (ICP) has been recently introduced^2^,^6^,^7^,^8^. ICP indicates the polarization of electrolytic concentrations at the anodic and cathodic side of a permselective membrane under d.c. bias. Typical behaviour is that the concentration becomes largely depleted at the anodic side (also known as an ion-depletion zone) and enriched at the cathodic side (also known as an ion-enrichment zone) in the case with a cation-selective membrane and vice versa^9^. In addition to nanoporous membranes^10^,^11^,^12^, bipolar electrodes^13^,^14^ and nanoporous particles^15^,^16^ have been reported for a new practical platform to generate ICP as well. Intensive researches have been conducted to explore its fundamental aspects such as the source of over-limiting current^17^,^18^,^19^, the vortex generation^20^,^21^,^22^ and related instability issues^23^ and its novel engineering applicability such as biological sample preparations^24^,^25^, energy saving mechanisms^26^ and electro-desalination applications^2^,^14^. Although ICP desalination method is unprecedentedly capable of extracting fresh water from highly saline, biologically infected or even heavy metal contaminated water, the necessity of a high external electrical power source^4^,^27^ is still the most critical hurdle to be overcome. Therefore, a new desalination mechanism that consumes the minimum level of energy is highly demanded.

In nature, mangrove trees that grow in saline water along the tropical or subtropical coasts are well known for their unique physiological responses to survive in highly saline environments without any external supply of mechanical, chemical or electrical powers^28^. The phenomenological observations of mangrove^28^ so far have found that the root is able to provide sap of 90% lower salinity than the external saline water by a reverse osmosis mechanism. Therefore, we are particularly interested in the ability of mangrove to exclude salt without any external energy. Motivated by this fact, we suggest a new spontaneous desalination mechanism by introducing the nanoscale electrokinetic and the hydrodynamic transportation of ions at the interfaces between the nanoporous structure and the saline environment. We visualize the ion movements that illustrate the new class of the ion-depletion phenomenon. By utilizing dry ionic hydrogel, the effective ionic flux through the hydrogel is induced by the capillarity of the hydrogel itself, instead of an external electrical bias, so that we name the currently exploited process as the capillarity ion concentration polarization (CICP). Osmo-poro-elastic process due to the swelling of the hydrogel^29^,^30^ is excluded in this work by a mechanical confinement^31^,^32^,^33^ within a microchannel to focus on the role of capillarity. Both analytical and numerical models are obtained to analyse the statics and dynamics of the spontaneous desalting mechanism. Since the mechanism is independent from any electrical power sources, it offers significant merits in terms of a power consumption and a stable operation compared with the conventional ICP mechanism. Therefore, CICP mechanism can be applied to power-free and small-scale (possibly portable) desalination/purification devices that would be particularly useful in remote/rural and disaster-stricken areas.

## Results

### Imbibition rates through the ionic hydrogel

**Figure 1:**
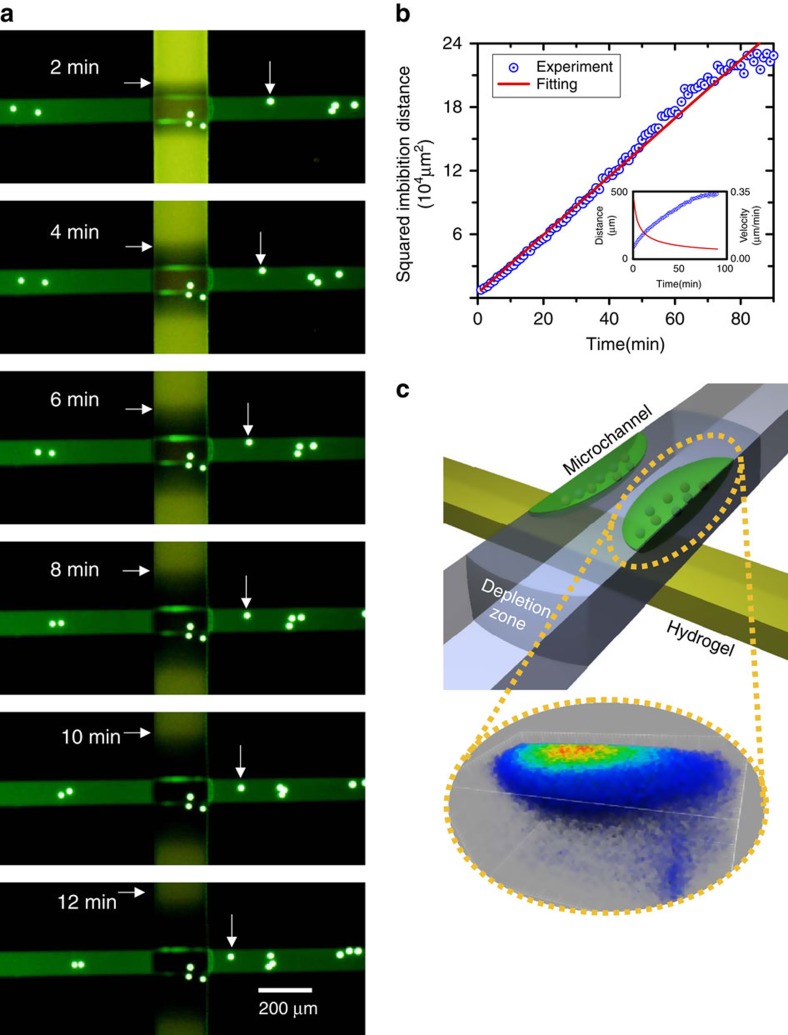
The formation of CICP zone. (**a**) The snapshots of a fluid flow driven by nanoporous hydrogel (see Supplementary Video 1) and (**b**) the plot of measured imbibition lengths as a function of time. The imbibition velocity is also plotted in the inset. (**c**) The confocal microscopic image of accumulating fluorescent dyes at the top corner of microchannel, confirming the formation of an ion-depletion zone at the bottom of microchannel.

The ionic hydrogel is capable of absorbing ambient aqueous solution by the capillarity. This phenomenon is critical to the presented CICP system, which utilizes the capillarity as a driving force for a liquid transportation. A hydrogel usually has an osmo-poro-elastic effect due to a significant swelling. However, the swelling has been reported to be minimized by a mechanical confinement^31^,^32^,^33^. In this work, the hydrogel swells within a microchannel and, thus, we are able to analyse the CICP phenomenon only by the capillarity effect. See Supplementary Note 1 for the swelling result either with or without mechanical confinements. As shown in Fig. 1a, there are two significant evidences of the imbibition by the capillarity. Micro-particles are injected near the hydrogel pad and their motions are captured at distinct time intervals. As indicated, their paths converge toward the hydrogel pad from both reservoirs in the centre-connection device, evidencing a mass sink at the centre. See Supplementary Video 1. In addition, the particle convergence in the end-connection device is also shown in Supplementary Note 2. The descriptions of the centre- and the end-connection device are given in the ‘Methods' section. Since there are no external driving forces (such as gravity or external electric/magnetic/acoustic field) other than the capillary force, the total volume that has disappeared (*V*_water_) is calculated by the speed of the particles. Since we confirm that there is no leakage at the bonding or hydrogel interfaces, the entire *V*_water_ should be transported into the hydrogel. Because the hydrogel has auto-fluorescence and it disappears when in contact with water molecules, the wetted volume of hydrogel (*V*_wet_) is able to be measured by the progression of an imbibition front. Therefore, the fraction of these two volumes (*V*_water_/*V*_wet_) is the same as the porosity of the hydrogel and it is calculated to be around 0.1, which would be used as one of the important parameters in the theoretical calculations.

Furthermore, the dynamic measurements of the imbibition length reveal that it grows as the square root of time as shown in Fig. 1b. This result is consistent with the classical Washburn's equation that describes the temporal evolution of the imbibition length in a porous medium^34^. Thus, the imbibition rate shown in the inset of Fig. 1b indicates that the capability of absorbing solution decreases as a function of time. This fact significantly affects CICP phenomenon as a restoration phase and would be discussed later on as well.

### The formation of the ion-depletion zone by CICP phenomenon

The conventional ICP phenomenon generated under an external d.c. bias results in significant concentration gradients at the anodic and cathodic sides of a permselective nanoporous membrane. Typical behaviour is that the ion concentration becomes extremely low at an anodic side (referred to as an ion-depletion zone) and enriches at a cathodic side (referred to as an ion-enrichment zone) in the case of a cation-selective membrane. Although the mechanism involves complex interactions of electric fields and electrokinetic flows^35^,^36^,^37^, the eye-catching fact in terms of engineering applications is that ICP can play as an electrical filter. This virtual barrier rejects the entrance of charged species into the ion-depletion zone, so that it can be utilized as a water desalination/purification mechanism^2^,^38^ and a biomolecular preconcentration mechanism^24^,^25^. Instead of an electrical bias, one can achieve the same ICP phenomena if another driving force pushes or pulls an electrolyte solution into the permselective membrane. Therefore, the capillarity is used in this work to initiate ICP phenomena. In this sense, the ion-depletion zone should also be formed in CICP system, which we confirm using a confocal microscope as shown in Fig. 1c. Since the driving force for a liquid flow due to the capillarity is much weaker than that due to an electrical bias in general, the depletion zone initially stays on the ionic hydrogel for a few hours (while the zone quickly expands in a conventional ICP system within a few seconds). A noticeable observation is that the fluorescent trackers are trapped at the top corners of a microchannel as shown in Fig. 1c. If there were no such ion-depletion zone, the trackers should have been accumulated at the bottom of the microchannel. In the meantime, the centre of the fluorescent cloud exhibits the brightest signal, since the flow converges into the hydrogel. Therefore, the confocal images with most of the fluorescent signals gathered at the corners of the microchannel provide strong evidences of CICP formation on the hydrogel pad.

### CICP as the spontaneous desalting mechanism

**Figure 2:**
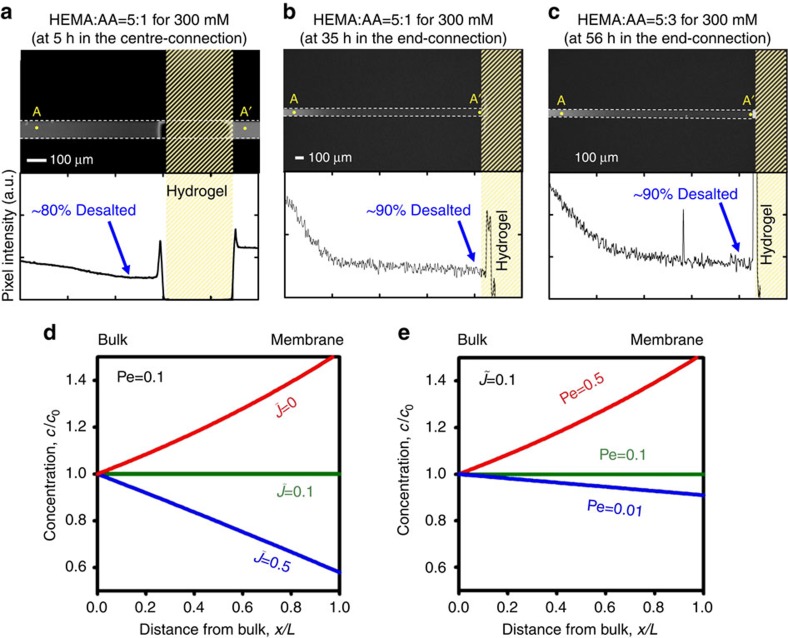
Spontaneous desalting by CICP. A fluorescent signal tracking through the microchannel with different compositions of the ionic hydrogel, (**a**) HEMA: AA=5:1 in the centre-connection device, (**b**) HEMA: AA=5:1 and (**c**) HEMA: AA=5:3 in the end-connection device. The plots of normalized concentration inside the ion-depletion zone with fixed (**d**) Pe at 0.1 and (**e**) ionic flux at 0.1.

Compared with the conventional ICP system, the formation of the ion-depletion zone in CICP system takes several hours since it relies only on the capillary force. The fluorescent microscopic images are captured as shown in Fig. 2a–c. Each snapshot is captured at different instances as indicated in the caption when the depletion zone expands in maximum. As shown in Fig. 2a, the fluorescent signal linearly decreases from the bulk solution as indicated at the line profile along A–A′. This is because the CICP is driven only by the capillarity, while the conventional ICP has a sudden drop and a flat concentration profile with fast electrokinetic vortices^18^,^19^,^36^. Before the measurement, the reference signals of various concentrations of fluorescent dye are obtained. See Supplementary Note 3. By comparing with the reference signal intensities, the dye molecules that infer the electrolyte concentration at the interface of hydrogel pad is expected to be removed with approximately 80%. Here, the ions are assumed to behave similar to fluorescent dyes. At least, the length of the depletion zone for ions and dye molecules are the same. This is confirmed by additional numerical simulations and experimental demonstrations. See Supplementary Note 4 for detailed explanation. Although Fig. 2a is conducted on the centre-connection device, the depletion zone asymmetrically expanded (that is, the depletion zone is rarely developed on the right side of the hydrogel). This is caused by an unavoidable residual flow from the level difference between the reservoirs. Detailed discussion would be given later on. To avoid the unwanted residual flow, the end-connection device is tested as shown in Fig. 2b,c. The dye concentration profiles have a similar linear drop and the removal efficiency is expected to be around 90%, which is similar to that of absorbed liquid by mangrove^28^. By comparing Fig. 2b with Fig. 2c, the duration of the depletion behaviour largely depends on the composition of the hydrogel. Increasing the volume fraction of charged group (acrylic acid (AA) in this work) provides the longer duration of CICP zone.

These behaviours, especially for a static case, are solved analytically. Under the assumption that the CICP system has an ideally cation-selective membrane with electroneutrality, a one-dimensional domain ignoring a surface effect and a quasi-equilibrium, the analytical solutions of the non-dimensional concentration (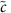) and electric potential (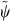) are given by


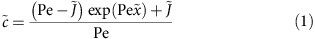


and


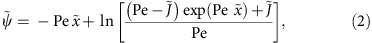


where 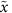 is the dimensionless spatial coordinate whose origin coincided with the bulk reservoir and 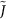 is the dimensionless ionic flux induced by the imbibition through the membrane.

Although CICP experiments are conducted without any electric power source, the electric interactions between ionic species should appear in terms of electro-migration. Pe is the Péclet number, which is the ratio of a convective transportation to a diffusive transportation (*uL*/*D* in this system). See the ‘Methods' section for full governing equations, boundary conditions and detailed derivations. These simple analytical solutions imply the most important parameters for enhancing the desalination efficiency near the membrane. The experimental results show that the electrolyte concentration is depleted near the membrane (that is, the concentration gradient is negative, d*c*/d*x*<0) so that 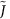 is greater than Pe, since 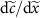 is equal to (Pe−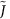) exp[Pe 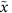]. Figure 2d,e show the concentration profile plots at the different physicochemical parameters, while Pe and 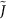 are fixed at 0.1 for each plot because the microfluidic device has Pe of ∼0.1 with *D*∼O(10^−9^) m^2^ s^−1^, *L* (the length of the microchannel)∼O(1) mm and *u* (a flow velocity due to an imbibition)∼O(10) μm min^−1^. The physical intuitions from the plots are such that higher 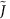 and lower Pe give a severer depletion. Since 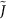 is proportional to the charge imbalance (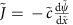, See equations (13) and (16) in the ‘Methods' section), the higher permselectivity would enhance the formation of the ion-depletion zone. This is also confirmed in Fig. 2b,c. By comparing the plots, the higher composition of the charged group (AA) leads to a stronger depletion behaviour which implies a longer duration. However, an excess amount of the charged group in the hydrogel results in a brittle structure so that one should tweak the composition as one's discretion. For the latter case, a diffusion limited system (that is, lower Pe) drives the depletion phenomenon more efficiently.

In Fig. 2a, the depletion zone propagates asymmetrically. An inevitable liquid level difference between each reservoir in the centre-connection device results in a residual flow over hydrogel pad. In the case of the residual flow from right to left (with the imbibition flow still converging to the centre from both reservoirs), the velocity of combined flow would increase at the right interface of the hydrogel and decrease at the left interface of the hydrogel. On the basis of the above theoretical analysis, low Pe would give a better depletion efficiency than high Pe. Since Pe is defined as a flow rate over a diffusion rate, there is a higher chance to have a stronger depletion zone at the left interface in Fig. 2a than the one at the right interface. See Supplementary Note 5 for a detailed description.

### The restoration phase by a diminished imbibition

**Figure 3:**
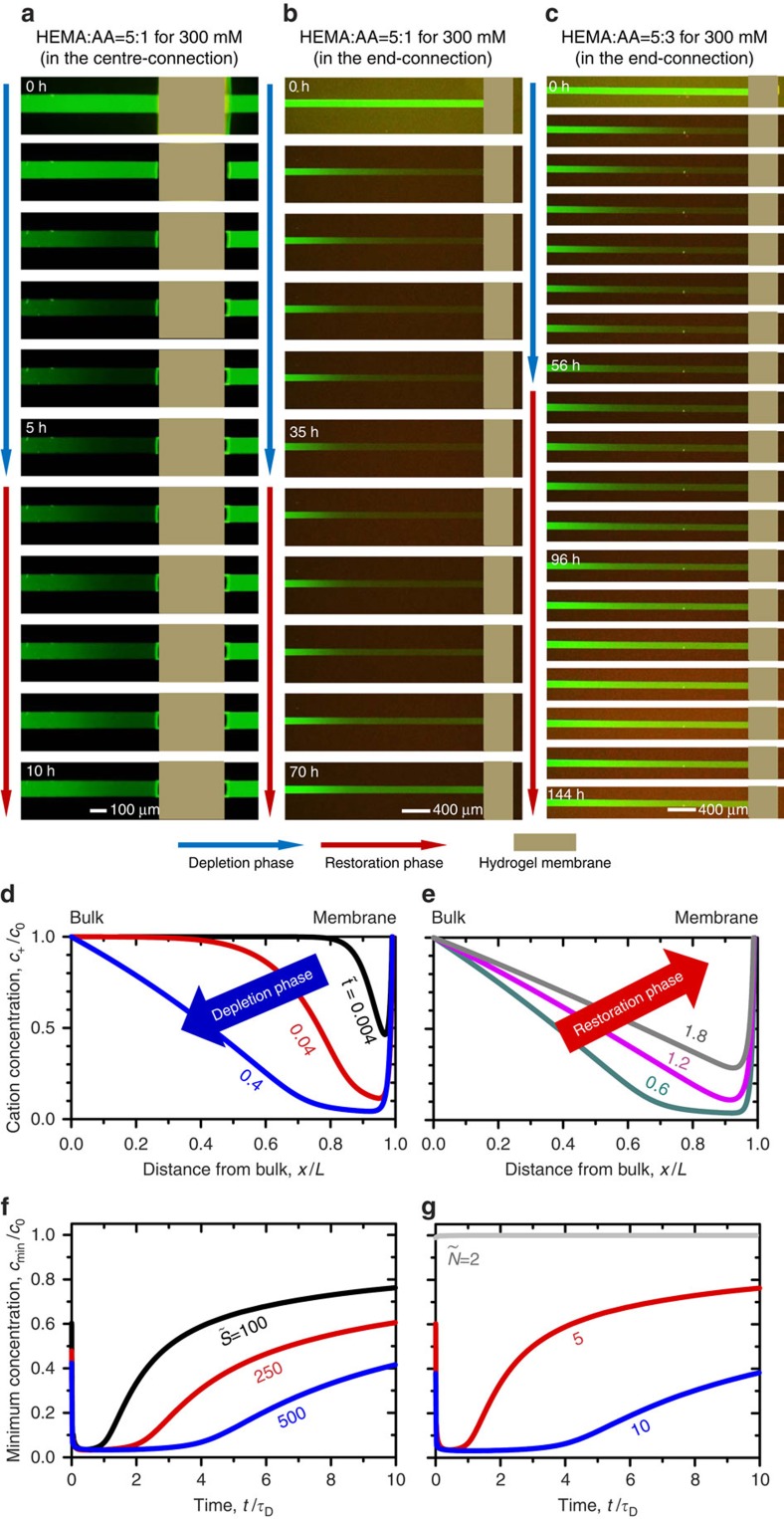
The depletion and the restoration phase of CICP. The dynamics of CICP system showing the transition from the depletion phase to the restoration phase with the different composition of the ionic hydrogel. (**a**) HEMA: AA=5:1 in the centre-connection device, (**b**) HEMA: AA=5:1 and (**c**) HEMA: AA=5:3 in the end-connection device. See Supplementary Videos 4, 5, 6 for each figure. Cation concentration profiles inside the CICP zone at (**d**) the depletion phase and (**e**) the restoration phase. See Supplementary Video 7. The minimum cation concentrations inside the ion-depletion zone with varying (**f**) imbibition parameters and (**g**) Donnan equilibrium concentrations.

The aforementioned depletion zone is formed and sustained for a few hours, depending on the composition of the ionic hydrogel and the geometry of the microchannel. However, the ion-depletion zone collapses and the concentration restores to the bulk concentration as shown in Fig. 3. See Supplementary Videos 4, 5, 6. This has never been observed in a conventional ICP phenomenon, since it continuously applies an external power. Note that the snapshots are captured every 1, 7 and 8 h in Fig. 3a–c, respectively. One interesting feature other than the appearance of the restoration phase is that the rate of the restoration phase is much slower than the rate of the depletion phase.

The transient analysis of CICP provides a clue for understanding the restoration phase. In CICP, the imbibition rate gradually decreases as a function of time as discussed above using the Washburn's equation^34^. Without loss of generality, the imbibition rate is expressed as


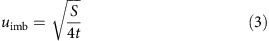


where *u*_imb_ is the imbibition velocity, *S* is the imbibition parameter and *t* is the time. As shown above, *u*_imb_ as the source of CICP is proportional to *t*^−0.5^, implying a high initial imbibition rate, but it should decay with time. Detailed descriptions are given in the ‘Methods' section.

Through the transient model, the dynamic changes of CICP layer are investigated. With chosen parameters in the ‘Methods' section, the depletion phase as depicted in Fig. 3d lasts for 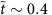 when the non-dimensional imbibition parameter 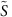 is 100 (normalized by *D*^2^*τ*_D_/*L*^2^). However, beyond this point, the phase transits to the restoration phase as shown in Fig. 3e because of the decreased imbibition rate. See Supplementary Video 7. These simulation results largely correspond to the experimental results in Fig. 3a–c. Although the imbibition rate decreases gradually in both the depletion and the restoration phase, the contribution of the imbibition in the depletion phase is still larger than the dissipation of ionic species induced by a diffusion. Briefly, the depletion phase initially appears (that is, the imbibition rate >the dissipation) and changes into the restoration phase (that is, the imbibition rate <the dissipation).

In Fig. 3f, the minimum cation concentrations inside the depletion zone are plotted with varying imbibition parameters, 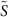, as a function of time. The parameter could be tuned by the absorbing capability of the ionic hydrogel. The imbibition parameter is defined by (*γ d*_cap_)/(4*Dη*) where *γ* is the surface tension, *d*_cap_ is the diameter of a capillary and *η* is the dynamic viscosity of the fluid. The increase of *γ* and *d*_cap_ or the decrease of *η* are the solutions to enhancing the imbibition rate. Although the Washburn's equation has been known to describe the imbibition through a single capillary tube, recent reports^39^,^40^,^41^ suggested that the functional form of the imbibition in the case of a capillary network also follows the Washburn's equation. Nevertheless, the transient analysis with arbitrary 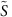 provides the insight for understanding the transition from the depletion phase to the restoration phase. Larger 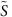 generates a higher imbibition rate, which is enough to overcome the dissipation so that the depletion phase sustains longer. Moreover, the ionic hydrogel of a higher charges has more sustainable depletion phase by comparing Fig. 3b,c. The hydrogel used in Fig. 3c has the higher composition of the charged group, AA. In this theoretical model, the non-dimensional Donnan equilibrium concentration (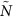) is related to the intrinsic charge of the membrane. The membrane possessing a higher charge generates a higher ionic flux through the membrane, so that the depletion phase can last longer as shown in Fig. 3g with fixed 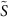 at 100.

## Discussion

In this study, we propose a new ICP phenomenon driven by capillarity. Instead of an external electric field, the inherent capillarity of nanoporous network drives a permselective ion transportation through the nanoporous hydrogel network, leading to the ion-depletion zone near the ionic hydrogel. Although the state-of-the-art desalination method utilizing a size exclusion, a thermal or electrical energy have remarkably advanced, the high level of energy requirements has hampered the development of a small-scale desalination/purification system. The fact that the CICP mechanism is working without any electrical power source offers significant merits in terms of the power consumption and the stable operation compared with the conventional electrokinetic ICP mechanism and other state-of-the-art large-scale desalination methods. Therefore, the present spontaneous desalting mechanism can lead to a power-free desalination system that is significantly useful in remote/rural and disaster-stricken areas. To achieve such a system, maintaining a quiescent fluid near the nanoporous membrane (that is, low Péclet number), enhancing the intrinsic surface charge of the membrane (that is, high ionic flux) and sustaining an imbibition at a certain level (that is, preventing restoration phase) are strongly required.

## Methods

### CICP device fabrication

**Figure 4:**
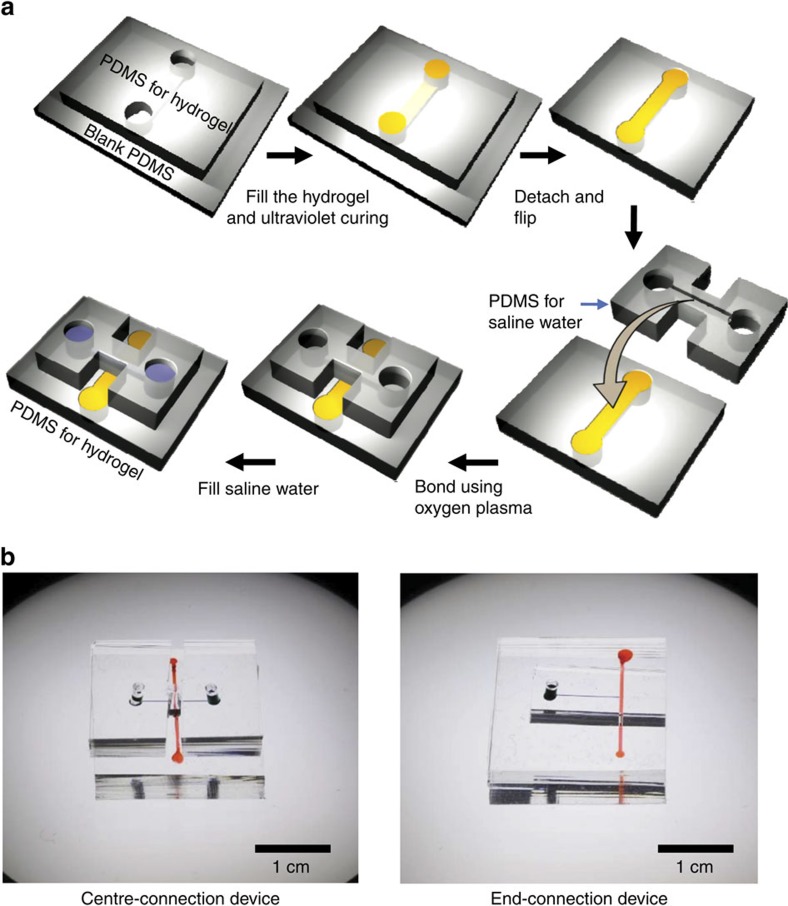
A micro/nanofluidic CICP device. (**a**) The fabrication processes of CICP system. (**b**) The photo of assembled CICP devices of the centre-connection device and the end-connection device.

The main building block of the CICP system is polydimethyl-siloxane (PDMS) material as schematically shown in Fig. 4. The final device consists of two layers of PDMS blocks. The bottom block is for a microchannel filled with the ionic hydrogel and the top block is for a microchannel to inject the saline water sample. Silicon masters are prepared using SU8 photoresist with the microchannel dimension of 400 μm (width) × 50 μm (depth) × 16 mm (length) for bottom PDMS block and 100 μm (width) × 15 μm (depth) × 10 mm (length) for top PDMS block. The PDMS blocks are fabricated as follows. The PDMS material (Sylgard 184 Silicone elastomer kit, Dow Corning, USA) mixed with a curing agent at 10:1 ratio is poured on the silicon masters that have desirable microchannel patterns. Then the polymer solution is cured in an oven for 4 h at 75 °C.

Detailed fabrication process from the first schematic is as follows. First, only the top PDMS for hydrogel mould is treated with oxygen plasma (CUTE-MP, Femto Science, Korea) for adhesion of the hydrogel and attached to a blank bottom PDMS piece. Then the microchannel is filled with a hydrogel precursor material consisting of 2-hydroxyethyl methacrylate (HEMA), acrylic acid (AA), a crosslinker (ethylene glycol dimethacrylate (EGDMA)) and a photoinitiator, (2,2-dimethoxy-2 phenyl-acetophenone (DMPA)) at the weight ratio of 32.51: 6: 0.41: 1.25 (named 5:1 hydrogel) or 32.51: 18: 0.41: 1.25 (named 5:3 hydrogel)^12^. The ratio of HEMA to AA would have a significant effect on CICP phenomenon as discussed. All the materials are purchased from Sigma-Aldrich. Ultraviolet irradiation for 8 min at the dose of 225 mW cm^−2^ (WUV-L50, Daihan Scientific, Korea) polymerizes the hydrogel inside the microchannel and then the top PDMS block is detached from the bottom blank PDMS piece. The hydrogel inside the microchannel is initially brittle and stiff (dry state) but the hydrogel absorbs a solution so that the portion of hydrogel becomes flexible and tough as the CICP proceeded. See Supplementary Video 8 for demonstrating only the water-absorbed portion is flexible and tough. Finally the flipped PDMS block for hydrogel and the PDMS for saline water samples are irreversibly bonded with oxygen plasma treatment. Assembled devices placed on glass slide are shown in Fig. 4b. There are two distinct designs, a ‘centre-connection device' and an ‘end-connection device'. The centre-connection device is designed for easiness of facilitating the experiment such as sample filling and flushing, while a residual flow is able to be completely eliminated in the end-connection device.

The microchannel inside top PDMS block is filled with KCl electrolyte solution at the concentration of 300 mM mixed with a fluorescent dye (Alexa 488, Sigma Aldrich, USA) as a tracker of the ion movements and negatively charged micro-particles (*d*=5 μm, Invitrogen, USA) for flow field tracking. KCl is used for a simple theoretical interpretation by setting the same diffusivity of cation and anion and the concentration of 300 mM is the average salinity of brackish water.

### Experimental setup

Compared with the conventional ICP process, any external voltage source is no longer required for the present CICP device. Instead, the total experimental time takes from a few hours to a few days depending on the device (for example, a conventional ICP occurs within a few seconds) so that the images of flow and concentration changes are captured every 2 min for a few days using a commercial computer software (CellSens, Olympus, Japan) and an inverted fluorescent microscope (IX 53, Olympus, Japan). The location where the hydrogel channel and the sample channel crossed is intensively analysed. A confocal microscope (FV1200, Olympus, Japan) is used for the three-dimensional concentration profiling.

### Theoretical domain descriptions with governing equations

The numerical and analytical analyses are conducted for the following domain where CICP occurs. A microchannel with length *L* is filled with 1:1 electrolyte solution. One side of the microchannel opens to a well-mixed reservoir with bulk concentration, *c*_0_ (at *x*=0), while the other side contacts an ideal cation-selective surface (at *x*=*L*). Assuming that the ideal permselectivity allows us to avoid complexity in describing the transport phenomena inside the membrane^17^,^22^,^23^,^42^,^43^, the domain from the reservoir to the membrane surface becomes the region of interest to be theoretically investigated. Since the streaming potential generated by the imbibition through the membrane is negligible, the effects of a surface conduction and an electro-osmotic flow on the CICP are excluded. Therefore, the domain is simplified as a one-dimensional configuration.

In general, the transport of ionic species along the *x* axis is governed by the Nernst–Planck equation,


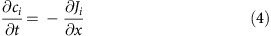


where *t* is the time, *c*_*i*_ is the concentration of *i*-th species and *J*_*i*_ is the ionic molar flux of the *i*-th species along the *x* axis. In accordance with transport mechanisms^44^, the ionic flux, *J*_*i*_ is given by


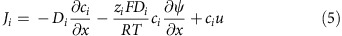


where *D*_*i*_ is the diffusivity of *i*-th species, *F* is the Faraday constant, *R* is the gas constant, *T* is the absolute temperature, *ψ* is the electric potential and *u* is the flow velocity of *x* direction. In equation (5), each term on the right-hand side corresponds to the transport mechanism due to a diffusion, an electro-migration and a convection, respectively. Although CICP experiments are conducted without any electric power source, the term of the electro-migration should be considered to describe the electric interactions between ionic species. Thus, the interactions are explained by the Poisson equation,


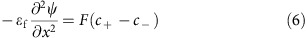


where *ɛ*_f_ is the electrical permittivity of the electrolyte solution, and *c*_+_ and *c*_−_ are the concentrations of cation and anion, respectively. The convective component of the ionic flux is usually obtained by solving the Stokes equations and the continuity equation with coupled partial differential equations (4)–(6), 24, 25. However, in this work, we adopt the simplified approach suggested by Dhopeshwarkar *et al*.^45^ to avoid mathematical complication. In their work, the driving force to generate ICP phenomena was the difference of applied electric potentials through the microchannel–nanochannel–microchannel system. However, our system is a microchannel–nanochannel connected system so that the theoretical domain is different and the imbibition through the membrane drives the ICP phenomena. Thus, the domain is reduced to a microchannel part rather than a whole microchannel–nanochannel connected system, while the domain of Dhopeshwarkar *et al*. analysed a whole microchannel–nanochannel–microchannel system. Since the effective convective transport is induced by the imbibition into the membrane, the flow velocity in the microchannel (*u*) is related to the imbibition rate (*u*_imb_) as


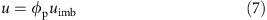


where *ϕ*_p_ is the porosity of the membrane, which is assumed to consist of a bundle of cylindrical capillaries. Equation (7) is derived from the fact that the flow rate in the whole system should be uniform. This imbibition is the driving force for CICP, while the driving force of conventional ICP platforms is usually the difference of applied electric potentials.

Detailed boundary conditions at the reservoir bulk (*x*=0) and the cation-selective surface (*x*=*L*) to describe the CICP would be discussed in the following sections.

### The derivation of analytical solutions at the steady state

Since each ionic flux of the *i*-th species is constant over the domain in the steady state, the Nernst–Planck equation is reduced to simplified balance equations as


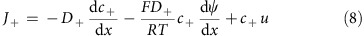


and


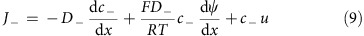


for 1:1 electrolyte solution. For simplicity, thin double layers and electroneutrality (*c*_+_=*c*_−_=*c*) are assumed in the microchannel as the domain. In addition, the diffusivity of each ionic species is fixed to the same value, (*D*_*+*_=*D*_−_=*D*). For the ideal cation-selective surface, no anion flux exists over the microchannel. As a result, the above equations are rewritten as


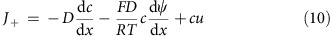


and


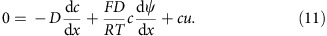


Combining equations (10) and (11) yields


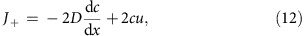


while subtracting equation (11) from equation (10) yields


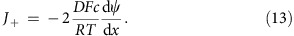


The equations (12) and (13) are further simplified by non-dimensionalization with


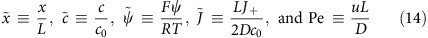


where Pe is known as the Péclet number, the ratio of a convective transport rate and a diffusive transport rate. Consequently, the non-dimensional forms of equations (12) and (13) are,


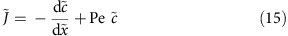


and


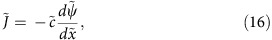


respectively. Because 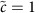 and 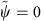 at 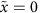, equations (15) and (16) can be integrated under constant 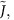 which is induced by the imbibition through the membrane. Therefore, the following analytical solutions are obtained with respect to the concentration and the electric potential inside CICP layer as


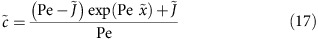


and


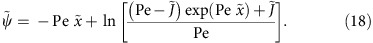


### The formulation of transient model

The imbibition into a capillary tube is described by Washburn's equation^34^ that is


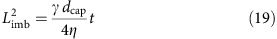


where *L*_imb_ is imbibition length, *γ* is the surface tension of water/capillary wall/air interface and *d*_cap_ is the diameter of capillary. By differentiating equation (19), the imbibition rate is obtained as


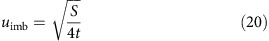


where *S* is defined by *γ d*_cap_/4*η*. Because *u*_imb_ as the source of CICP is proportional to *t*^−0.5^, the thickness of CICP layer induced by imbibition into the membrane would be decreased as time passed.

The spatiotemporal concentration profile inside the CICP layer is obtained by the following non-dimensional forms of equations (5) and (6) with appropriate boundary conditions,


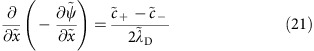


and


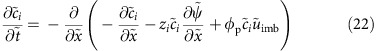


where the characteristic scales of *c*_*i*_, *ψ* and *x* are denoted in equation (14), the characteristic time is chosen as the diffusion time scale, *τ*_D_ defined as *L*^2^/*D*, and the characteristic velocity is set to be *D*/*L*. In equation (21), 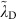 is the non-dimensional Debye length defined as


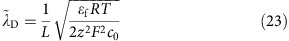


for *z*:*z* electrolyte solution. At the reservoir bulk (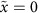), the following boundary conditions should be satisfied,


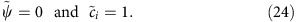


At the ideally cation-selective surface (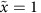), the cation flux through the ionic hydrogel pad is generated only by the imbibition,


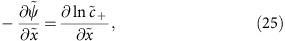


and the permselectivity is described by


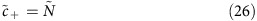


and


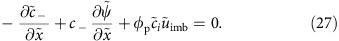


Equation (25) implies that the gradient of electrochemical potential (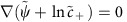) would be zero, so that only a convective transportation into the membrane contributes to form the CICP layer. The Donnan equilibrium concentration^17^,^22^ inside the nanoporous membrane is described by equations (26) and (27), which impedes that anion flux into the membrane should be zero due to the ideal cation selectivity. Equations (21) and (22) with boundary conditions of equations (24)–(27), 51, 52, 53 are solved numerically.

### Numerical method

Numerical simulations are conducted by COMSOL Mutiphysics 4.4, a commercial finite element method tool, under the one-dimensional domain and the transient analysis mode. The domain is discretized into 1,000 elements, with finer meshes in the proximity to the membrane surface. To carry out a qualitative analysis for CICP system, we assume that the diffusion time scale (*τ*_D_) and the porosity (*ϕ*_p_) are 500 s and 0.1, respectively. In addition, the non-dimensional Debye length and Donnan equilibrium concentration are set to be 0.01 and 5. From Washburn's equation^34^, the imbibition rate is infinite at *t*=0 s so that the initial conditions cannot be defined. To avoid this problem, a transient analysis is conducted from *t*=1 s rather than from *t*=0 s. The time step and the termination time are set to be 1 and 3,600 s, respectively.

## Additional information

**How to cite this article:** Park, S. *et al*. Capillarity ion concentration polarization as spontaneous desalting mechanism. *Nat. Commun.* 7:11223 doi: 10.1038/ncomms11223 (2016).

## Supplementary Material

Supplementary Information Supplementary Figures 1-7, Supplementary Tables 1-2, Supplementary Notes 1-5 and Supplementary References

Supplementary Movie 1 Particle converging in the center connection device.

Supplementary Movie 2 Particle converging in the end connection device.

Supplementary Movie 3 CICP operation with DI and 5:1 hydrogel in the end connection device.

Supplementary Movie 4 CICP operation with 300 mM sample and 5:1 hydrogel in the center connection device.

Supplementary Movie 5 CICP operation with 300 mM sample and 5:1 hydrogel in the end connection device.

Supplementary Movie 6 CICP operation with 300 mM sample and 5:3 hydrogel in the end connection device.

Supplementary Movie 7 Transient analysis of CICP operation.

Supplementary Movie 8 The mechanical stiffness of swollen hydrogel.
