# Supplementary material for: Capillarity ion concentration polarization as spontaneous desalting mechanism
Source: Nat Commun. 2016 Apr 1;7:11223. doi: 10.1038/ncomms11223 (PMC4822007; doi:10.1038/ncomms11223)
Supplement: Supplementary Information — Supplementary Figures 1-7, Supplementary Tables 1-2, Supplementary Notes 1-5 and Supplementary References [file ncomms11223-s1.pdf]

Supplementary Materials

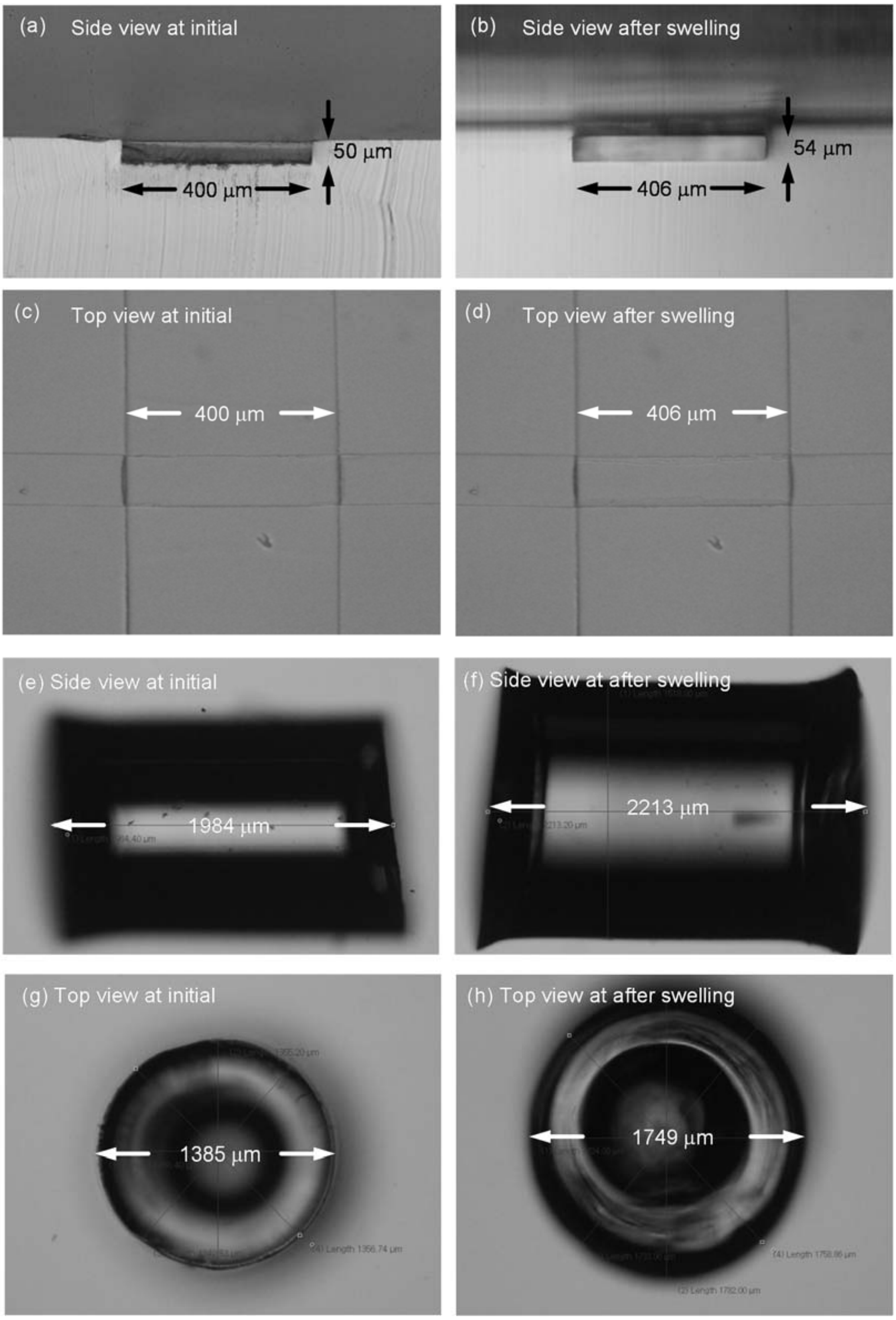

Supplementary Figure 1. The microscopic images of the hydrogel before and after swelling. The hydrogel swelled (a) ~ (d) with a mechanical confinement within a microchannel and (e) ~ (h) freely without a mechanical confinement.

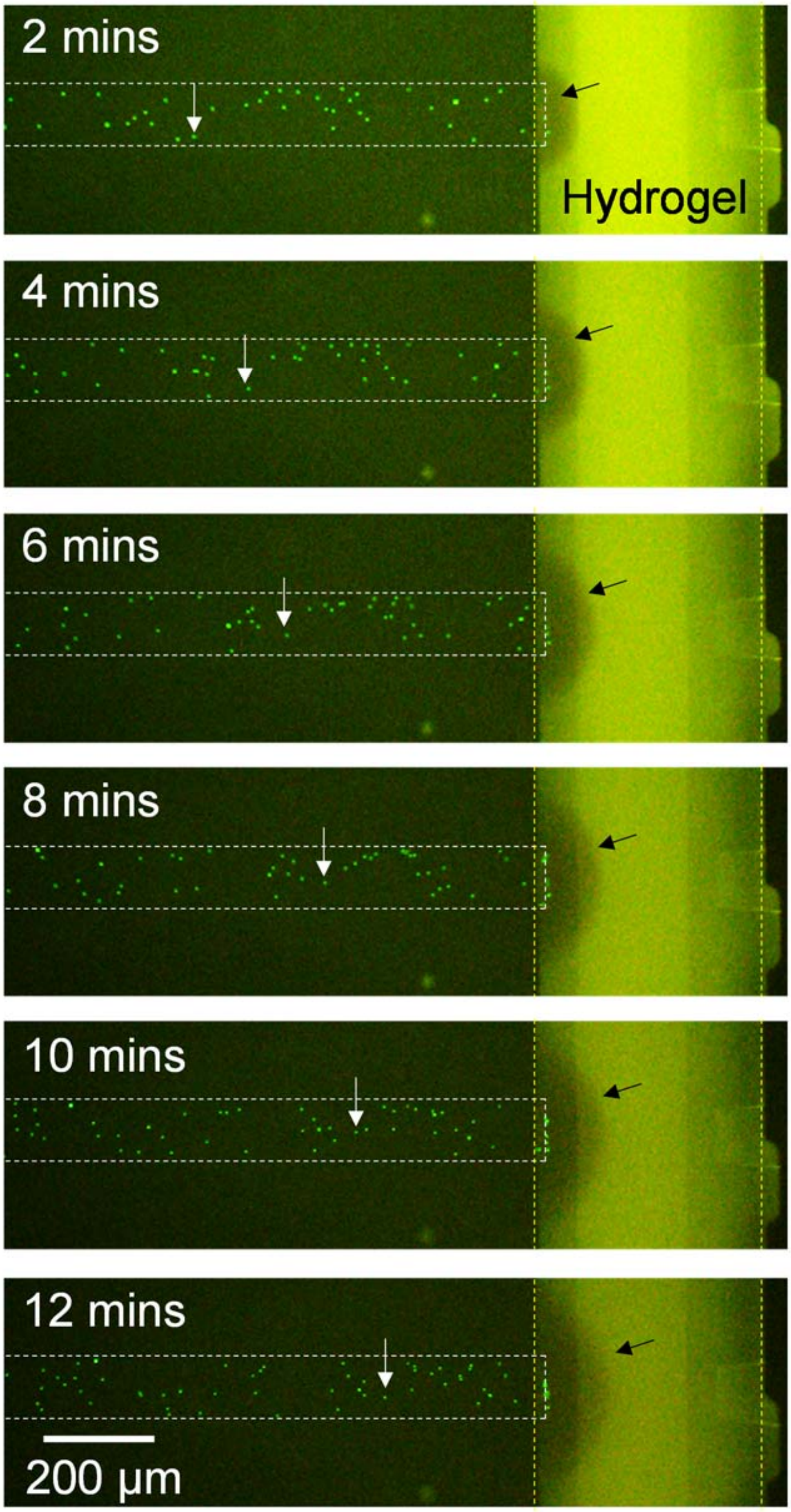

Supplementary Figure 2. The snapshots of the microparticle movements toward the ionic hydrogel.

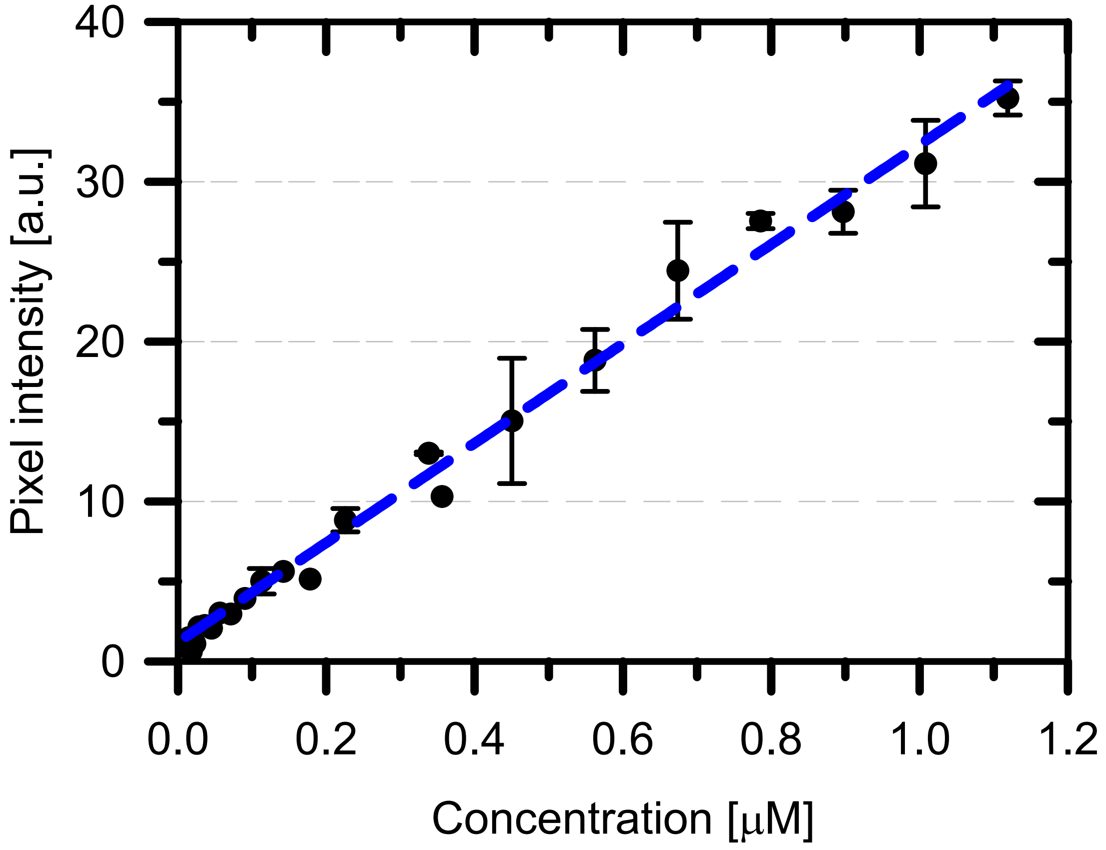

Supplementary Figure 3. Reference fluorescent signal as a function of the dye concentrations.

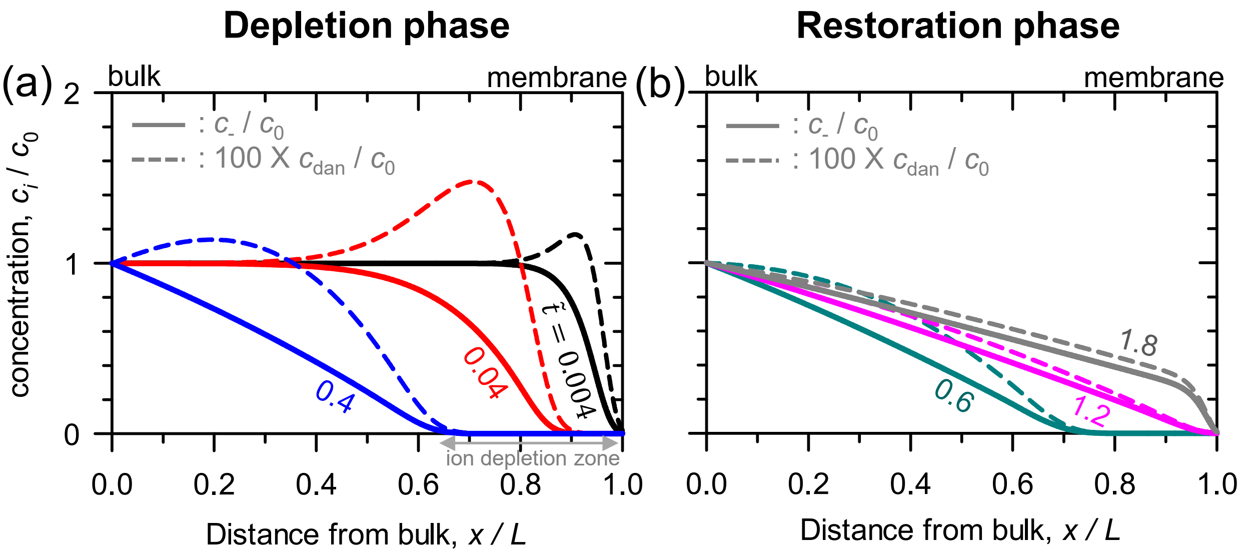

Supplementary Figure 4. Numerical simulation of CICP dynamics with diluted dyes. (a) The depletion phase and (b) the restoration phase with the conditions for electrolyte as c_+, c_- >> c_{dcat}, c_{dan}.

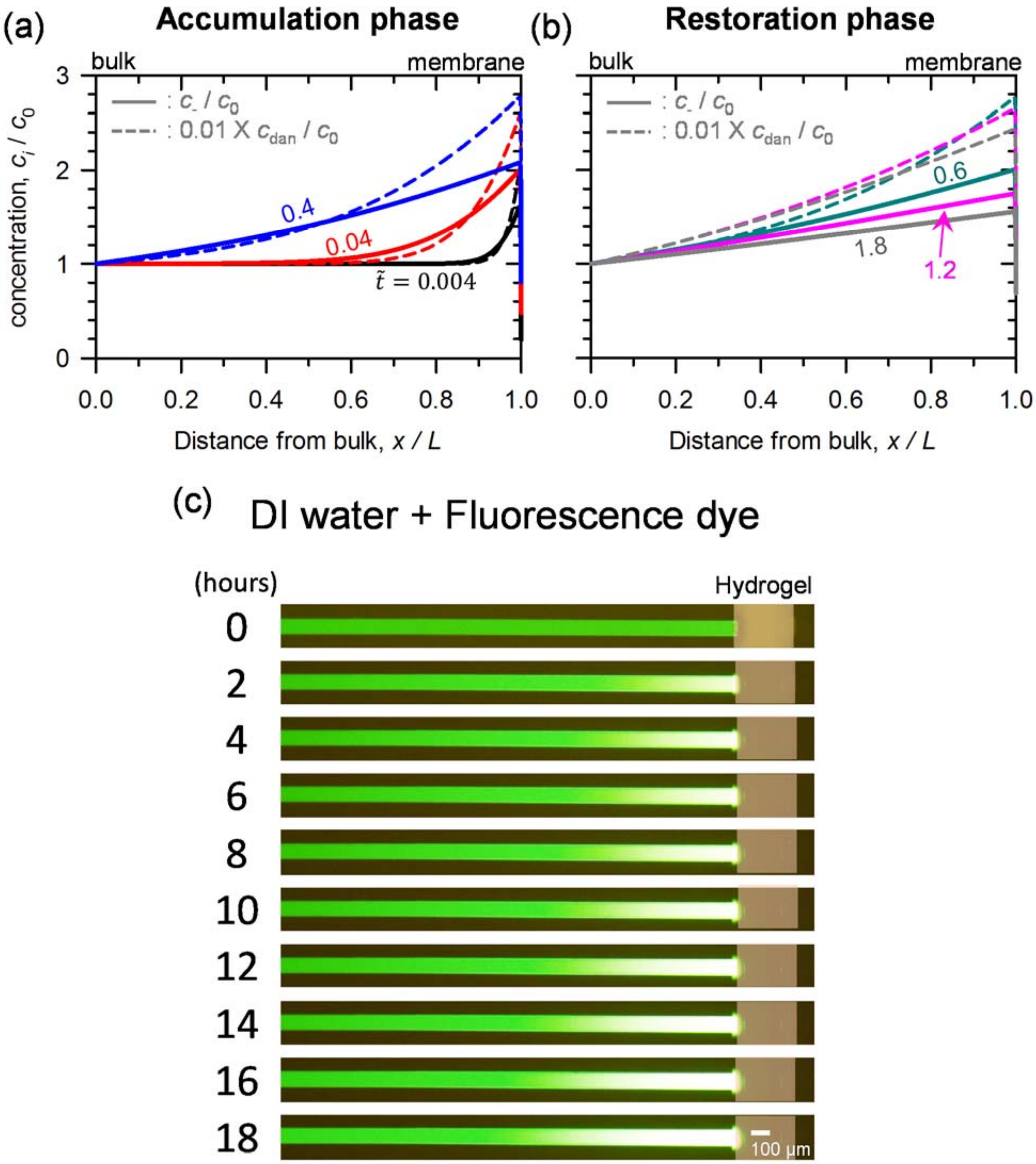

Supplementary Figure 5. Numerical simulation and experimental results of CICP dynamics with diluted solutions. (a) The accumulation phase and (b) the restoration phase with the conditions for electrolyte as c_+, $c_- \ll c_{dcat}$, c_{dan}. (c) The experimental verification for this condition of the electrolyte concentration. See Supplementary Video 3.

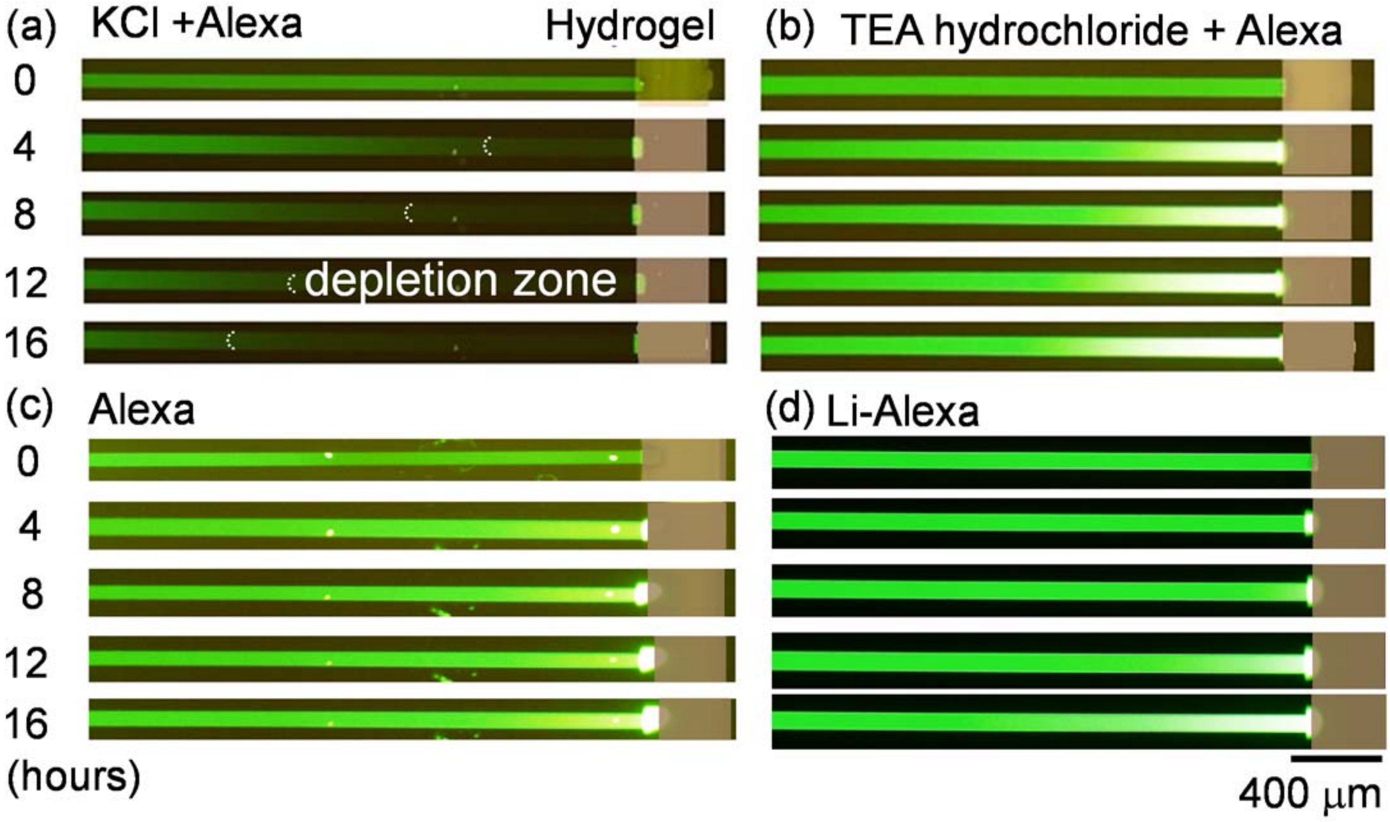

Supplementary Figure 6. The CICP operation with various electrolytes. The electrolytes are (a) #1 (KCl), (b) #2 (TEA hydrochloride), (c) #3 (Alexa), (d) #4 (Li-Alexa) and (e) #5 (LiCl).

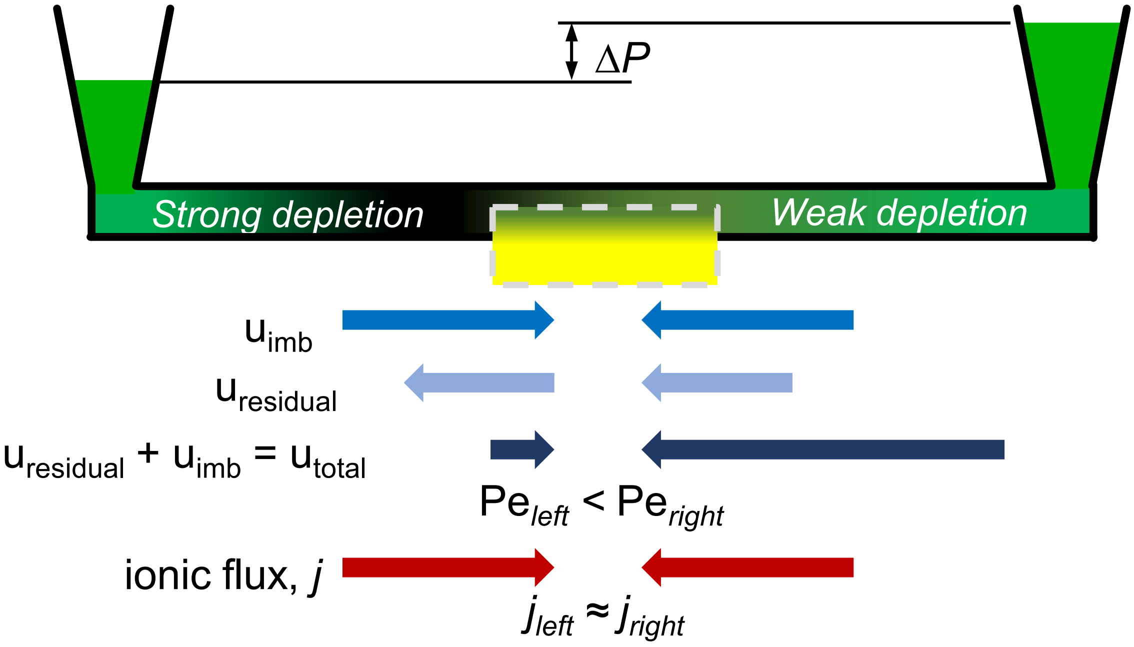

Supplementary Figure 7. The schematic diagram of total fluid flow under the consideration of an external residual flow by the level difference between reservoirs.

no.	Electrolyte	Major cation	Major anion	Fluorescent tracker	Size comparison
#1	DI + KCl 300 mM + Alexa 1 uM	K^+	Cl^-	Alexa$^-$	Li^+, K^+, Cl^- << TEA^+ , Alexa$^-$
#2	DI + TEA hydrochloride 3 mM + Alexa 1 uM	TEA^+	Cl^-	Alexa$^-$	
#3	DI + Alexa 1 uM	TEA^+	Alexa$^-$	Alexa$^-$	
#4	DI + Li-Alexa 1 uM	Li^+	Alexa$^-$	Alexa$^-$	
#5	DI + LiCl 300 mM + Alexa 1 uM	Li^+	Cl^-	Alexa$^-$	

Supplementary Table 1. The electrolytes used for the tests. For simplicity, the cation and the anion of Alexa 488 are denoted as TEA^+ and Alexa$^-$, respectively. TEA is triethylamine. Depending on a relative concentration, major cation and major anion are selected as shown. #1 and #2 have the same anion and #2 and #3 have the same cation. #4 and #5 are added for confirming that both conditions (small size of major cation and major anion) should be satisfied to generate the ion depletion region.

no.	Electrolyte	Counter-ion size	Co-ion size	CICP phenomena
#1	DI + KCl 300 mM + Alexa 1 uM	small	small	depletion
#2	DI + TEA hydrochloride 3 mM + Alexa 1 uM	large	small	accumulation
#3	DI + Alexa 1 uM	large	large	accumulation
#4	DI + Li-Alexa 1 uM	small	large	accumulation
#5	DI + LiCl 300 mM + Alexa 1 uM	small	small	depletion

Supplementary Table 2. The conditions for the generation of ion depletion region in CICP system.

Supplementary Note 1. A measurement of hydrogel swelling. A hydrogel is known to have significant swelling property in the case of contacting water molecule. However, in order to focus on the capillarity as the only driving force of the perm-selective ion transportation, we would like to suppress the swelling using a confinement within the microchannel network. The free energy of osmo-poro-elasticity plays a key role to swell the hydrogel[1], but it was reported that the swelling could be suppressed by an external mechanical constraint such as a confinement within a microchannel[2, 3]. As shown in Supplementary Figure 1, microscopic images of hydrogel ((a)~(d) within a confinement by a microchannel and (e)~(h) without the confinement) are captured at the initial stage and after swelling. The volume of hydrogel is expanded less than 10% with the confinement (the volume expanded from 16mm × 400 μm × 50 μm to 16mm × 406 μm × 54 μm) and 77% without the confinement (the volume expanded from π (692 μm)^2 × 1984 μm to π (874 μm)^2 × 2213 μm). Based on this measurement, the osmo-poro-elastic effect is successfully suppressed in this work.

Supplementary Note 2. Particle convergence toward the hydrogel in the end-connection device. The particle converging experiment that shows the imbibition into the hydrogel is also demonstrated in the end-connection device. As shown in Supplementary Figure 2, microparticles of diameter = 1 μm move toward the hydrogel and the imbibition front gradually expands as a function of time. See Supplementary Video 2.

Supplementary Note 3. Concentration measurement from reference fluorescent signal. A microchannel which has the same dimensions as used in the experiments is bonded to the glass to measure the reference intensity of florescence dye. The fluorescent dye (Alexa 488) is diluted with DI water at various concentrations. Each florescence dye solution is injected into the microchannel. Then the microscopic image of each solution is captured and analyzed by a computer software (CellSens, ImageJ). Imaging conditions are identical for every measurement. A plot of pixel intensity as a function of the concentration of fluorescent dye is obtained as shown in Supplementary Figure 3. Then the unknown concentration can be determined using the plot by an interpolation.

Supplementary Note 4. Theoretical and experimental analysis for the behavior of fluorescent dye. Typically, ionic fluorescent dyes such as sulforhodamine, Alexa, and Fluorescein have been used for visualizing the behavior of background electrolyte in microfluidics under an assumption that the dye would behave with the electrolyte. However, the assumption could be concerned, because the dye would electrostatically interact with the cations and anions dissolved in the electrolyte. In order to clear up the concern, we conduct the numerical simulations and experimental validations for the case of electrolyte/ionic fluorescent dye mixture.

In the mixture, there are four kinds of ionic species; cation (K^+), anion (Cl^-), dye cation ($C_6H_{16}N^+$, *dcat*), and dye anion ($C_{27}H_{12}N_2O_{11}S_2F_4^-$, *dan*) whose transport phenomena are governed by the Nernst-Planck equations. Since the diffusivity and the electrophoretic mobility of dye ions are different from the ions of electrolyte, the equation should be

$$\frac{\partial \tilde{c}_i}{\partial \tilde{t}} = -\frac{\partial}{\partial \tilde{x}}\left(-\tilde{D}_i \frac{\partial \tilde{c}_i}{\partial \tilde{x}} - z_i \tilde{\mu}_i \tilde{c}_i \frac{\partial \tilde{\psi}}{\partial \tilde{x}} + \phi_p \tilde{c}_i \tilde{u}_{imb} \right). \tag{1}$$

In the above equation, $\tilde{D}_i$ is dimensionless diffusivity and $\tilde{\mu}_i$ is dimensionless electrophoretic mobility of each ionic species. The both mobility of dye cation and dye anion are normalized by the value of K^+ and are set to be 0.5 which correspond with the value of Alexa 488[4]. The Poisson equation is also changed into

$$\frac{\partial}{\partial \tilde{x}}\left(-\frac{\partial \tilde{\psi}}{\partial \tilde{x}} \right) = \frac{\tilde{c}_+ - \tilde{c}_- + \tilde{c}_{dcat} - \tilde{c}_{dan}}{2\tilde{\lambda}_D} \tag{2}$$

where $\tilde{c}_{dcat}$ is the dimensionless concentration of dye cation and $\tilde{c}_{dan}$ is the dimensionless concentration of dye anion. Utilizing the Poisson equation, the electrostatic interactions between the dye molecules and the electrolyte can be simulated. At the reservoir bulk, both dye

cation and dye anion hold the reservoir concentration. At the ideal cation-selective surface, the flux of dye anion should be zero due to the permselectivity. In addition, we impose that the flux of dye cation is also zero, because it cannot penetrate through the membrane due to the size-selectivity.

Through the above formulations, we obtain numerical results as shown in Supplementary Figure 4 for the conditions of small amount of dyes in high concentrated electrolyte (c_+, c_- >> c_{dcat}, c_{dan}) and Supplementary Figure 5 for the conditions of minimal amount of electrolyte with small amount of dyes (c_+, c_- << c_{dcat}, c_{dan}). Supplementary Figure 4 is the typical situation in CICP experiment as well as usual microfluidic experiment. The dimensionless reservoir concentration of dye is set to be 1% of electrolyte concentration. Since only dye anions are detected as a fluorescent signal, we plot the behaviors of anion and dye anion. As shown in the Supplementary Figure 4(a), the dye anion is able to properly represent the length of the ion depletion zone in which the concentration is nearly zero. For example, the length of depletion zones (where the concentration value is almost zero) are about 0.3 regardless of c_- and c_{dan} at $\widetilde{t}$ = 0.4. While the dye accumulates right outside the depletion zone, this could be ignored because the maximum accumulation ratio is below 1.5 of which value is insensitive to detect. In the restoration phase as shown in Supplementary Figure 4(b), the dye anion still represents the depletion zone as well.

In Supplementary Figure 5, the behaviors are plotted for the case that the concentration of KCl is much less than the concentration of dye (*i.e.* c_+, c_- << c_{dcat}, c_{dan}). The dimensionless dye concentration is set to be 10,000% of the dimensionless KCl concentration. Comparing to Supplementary Figure 4(a), only accumulation phase is simulated instead of the depletion phase. Since the dye cations (acted as majority carriers) cannot pass through the membrane due to the size-selectivity so that they start to accumulate in front of the membrane. Thus, the

generation of the depletion zone by the imbibition of majority carriers would be impossible. After a while, the restoration phase would start as shown in Supplementary Figure 5(b). These results are well-matched with the experiment as shown in Supplementary Figure 5(c). In this experiment, the sample solution of 1.1 μM Alexa dye in DI water is used and the test is carried in the end-connection device. Through the good-agreement between the theoretical and experimental analysis, we conclude that the dye molecules would qualitatively behave as the ions in this CICP system.

For further investigation, we conduct additional experiments with various types of dyes and buffer concentrations. The properties of used samples are summarized in Supplementary Table 1. In Supplementary Figure 6(a), KCl solution (#1) is driven to a cation selective hydrogel by the capillarity and the ion depletion zone is noteworthly generated. As the major cation (K^+) passes thorough the hydrogel membrane, the concentration of K^+ ion decreases near the membrane and the major anion (Cl^-) and the fluorescent tracker is also pushed away from the membrane because of the electrical neutrality. In Supplementary Figure 6(b), #2 has the same major anion with KCl solution but has larger cation than the pore size of the hydrogel. Thus the cations of #2 (here we denote as TEA^+) is unable to pass through hydrogel so that all of ions (TEA^+, $Alexa^-$ and Cl^-) are accumulated near the hydrogel. By comparing Supplementary Figure 6(a) to Supplementary Figure 6(b), we conclude that smaller major cation size is demanded for the generation of the ion depletion region. In Supplementary Figure 6(c), DI + Alexa solution (#3) has the same cation with #2 and also shows the accumulation, leading to a conclusion that the larger major cation is expelled from the imbibition. In Supplementary Figure 6(d), Li-Alexa (#4) has small major cation (Li^+) and large major anion ($Alexa^-$). Li^+ passes through the hydrogel membrane and remaining anions should have been pushed away to satisfy the electrical neutrality. However, because of the large size of anion, all ions of Li-

Alexa solution are accumulated near the hydrogel. Comparing Supplementary Figure 6(a) to Supplementary Figure 6(c) and 6(d), we conclude that small major anion size is also required for the generation of the ion depletion region. Lastly, Supplementary Figure (e) demonstrates that the ion depletion zone is properly generated with 300 mM LiCl solution which has both small major anions and cations.

Here we consider five electrolytes and conclude that “both conditions” should be satisfied for the generation of the ion depletion zone. The small major co- and counter-ion size are necessary conditions to generate the ion depletion zone but if one of the condition is missing, both ions are accumulated near the hydrogel membrane showing the accumulation phase (Supplementary Table 2).

Therefore, if one observed the ion depletion zone by tracking large fluorescent dye ions, this confirms that the small major counter-ions (here K^+) have penetrated through the hydrogel and the small major co-ions (here Cl^-) and fluorescent tracker are simultaneously pushed away from the hydrogel. Conclusively, in this work, the movement of dye molecule is able to infer the behavior of ions.

Supplementary Note 5. Asymmetric formation of the ion depletion zone in the center-connection device. While the depletion region is adequately developed at the left hand side in Figure 3(a), the fluorescent intensity that qualitatively represents an ion concentration remained unchanged at the right hand side. This unbalance mostly caused by an external residual flow, which is induced by an inevitable liquid level differences between two reservoirs. It is explained by the change of fluid flow caused by the external residual flow as shown in Supplementary Figure 7.

The direction of residual flow ($u_{residual}$) is right to left, while the imbibition flow (u_{imb}) symmetrically converges into the hydrogel. The combined flow (u_{total}), thus, results in high Pe on the right hand side. Through the analytical solution, the depletion region would be destroyed at the right hand side and maintains at the left hand side due to the lower Pe. In addition, the residual flow also affects to the duration time of depletion region. The residual flow carries ions from right to left so that the depletion region is replenished by the ions, leading to the short duration of the depletion zone in case of this center-connection device. This result is confirmed in Figure 3 in the main text.

Supplementary References

1. Shengqiang C, Zhigang S. Equations of state for ideal elastomeric gels. *Europhys Lett)* **97**, 34009 (2012).

2. Marcombe R, Cai S, Hong W, Zhao X, Lapusta Y, Suo Z. A theory of constrained swelling of a pH-sensitive hydrogel. *Soft Matter* **6**, 784-793 (2010).

3. Suzuki A, Hara T. Kinetics of one-dimensional swelling and shrinking of polymer gels under mechanical constraint. *J Chem Phys* **114**, 5012-5015 (2001).

4. Milanova D, Chambers RD, Bahga SS, Santiago JG. Electrophoretic mobility measurements of fluorescent dyes using on-chip capillary electrophoresis. *Electrophoresis* **32**, 3286-3294 (2011).
